# Effects of environmental relevant concentrations of acetochlor on growth, hematology, serum biochemistry and histopathology of Japanese quail

**DOI:** 10.1371/journal.pone.0306583

**Published:** 2024-09-20

**Authors:** Naveed Arshad, Sana Alam, Muhammad Rafay, Ghazala Jabeen, Kashif Hussain, Riaz Hussain, Muhammad Irfan Ullah, Mujahid Iqbal, Dalia Fouad, Farid Shokry Ataya

**Affiliations:** 1 Department of Forestry, Range and Wildlife Management, Islamia University of Bahawalpur, Pakistan; 2 Department of Zoology, The Islamia University of Bahawalpur, Pakistan; 3 Department of Zoology, Lahore College for Women University, Lahore, Pakistan; 4 Department of Pathobiology, Faculty of Veterinary and Animal Sciences, MNS University of Agriculture, Multan, Pakistan; 5 Department of Pathology, Faculty of Veterinary and Animal Sciences, The Islamia University of Bahawalpur, Pakistan; 6 Department of Pathobiology, Faculty Veterinary Sciences, Bahauddin Zakariya University, Multan, Pakistan; 7 Department of Pathology, Cholistan University of Veterinary and Animal Sciences, Bahawalpur, Pakistan; 8 College of Veterinary Medicine, Huazhong Agricultural University, Wuhan, China; 9 Department of Zoology, College of Science, King Saud University, Riyadh, Saudi Arabia; 10 Department of Biochemistry, College of Science, King Saud University, Riyadh, Saudi Arabia; University of Ibadan Faculty of Science, NIGERIA

## Abstract

Acetochlor is frequently applied to various food crops in agriculture sector, and long-term exposure can cause significant endocrine-disrupting effects in exposed animals including impacts on human health. This study aimed to evaluate the effects of acetochlor on the growth, hematology, serum biochemistry, and histopathological alterations in Japanese quail. Eighty male quail were obtained and divided into four groups (A-D) and given acetochlor orally for the period of 45 days. Group A was served as the control, while groups B, C, and D received 20mg/kg, 30mg/kg, and 40mg/kg acetochlor, respectively. The study found that Japanese quail administered higher doses of acetochlor exhibited reduced frequency of crowing and foam production. The results showed that increased concentrations of acetochlor led to adverse effects on the growth parameters of Japanese quail. Hematology analysis indicated that birds exposed to higher concentrations of acetochlor experienced a significant decrease in red blood cell count, hemoglobin concentration, hematocrit (HCT), mean corpuscular volume (MCV) and mean corpuscular hemoglobin concentration (MCHC), along with a significant increase in white blood cell count compared to the control group. Additionally, higher concentrations of acetochlor led to a significant increase in various serological indices including urea, creatinine, alanine aminotransferase (ALT), and aspartate aminotransferase (AST), while the values of total proteins, albumin, and plasma proteins declined. The histopathology results of treated Japanese quail exposed to higher concentrations of acetochlor showed a range of pathological lesions in the testes, heart, and brain. The study concluded that even low concentrations of acetochlor can cause slight to significant changes in Japanese quail, affecting their physical, hematological, histopathological and serum biochemical parameters.

## Introduction

The extensive use of synthetic pesticides has been implemented to enhance crop yields and increase agricultural productivity [1, 2]. With the growing demand for crops to feed the rapidly increasing global population, the utilization of agricultural chemicals has expanded [3, 4]. The high demand for food has led to an increased rate and quantity of pesticide use, which currently accounts for over 60% of all consumed pesticides [5, 6]. However, the use of pesticides especially herbicides can lead to the contamination of food and water sources, posing a threat to both humans and avian wildlife in numerous countries. As a result, birds have been utilized as environmental bio-monitors for several decades [7, 8]. Acetochlor is a commonly used herbicide to control broadleaf weeds and annual grasses in various crops [9]. It is known to be a persistent chemical that can be detected up to a year after its application. This herbicide is one of the most commonly detected substances in various environments, including sugarcane-growing areas in China and other Asian countries, where it has been found in 33.3% of water samples with a maximum residual concentration of 0.311 g/L [10]. The dietary LC50 (>4610 ppm) of acetochlor and oral LD50 (1260 mg/kg) of technical acetochlor [11, 12] for bobwhite quail has been estimated. It is recorded that the biodegradation of acetochlor in the natural environment is fairly slow (8 to 100 days) and low (33%) when applied at a concentration of 10 mg /kg in soil [13]. The peak concentrations (0.03–709.37 mg/kg) of acetochlor in riparian soil and residual concentrations (54.76 mg/kg) in maize land have been detected [14, 15].

Different herbicides prevent the growth of various broadleaf weeds via numerous mechanisms, such as the disruption and inhibition of photosynthesis, mitosis, root growth, leaf formation, enzyme functions and cell division [16]. Herbicides induce different disorders in broadleaf weeds and interfere with synthesis of various cellular pigments (DNA and proteins) and damage cell membranes [17, 18].

The blood and plasma of wild birds have been identified as suitable candidates for monitoring pesticide contamination in the environment, as they can be easily collected following bird trapping and provide a recent snapshot of pesticide exposure, enabling real-time monitoring of contamination levels [19–21]. It is noteworthy that exposure to pesticides is linked to a number of human diseases, including cancer, neurological diseases, hypertension, and obesity [22]. Further classification of acetochlor by the US Environmental Protection Agency (EPA) as a probable human carcinogen is concerning. Acetochlor exposure can activate the P450 enzyme system in rats, which then activates the carcinogenic intermediate dialkyl quinine imine, raising the risk of liver, stomach, and nasal cancer [23]. The frequent and persistent application of different pesticides causes several adverse impacts on both the environment and public health, such as an increased risk of abortion, infertility, congenital anomalies, and other health disorders due occupational exposure [24]. The persistent application of herbicide has resulted in the common detection of acetochlor in water and soil and is associated with induction of melanoma, pancreatic cancer, lung cancer, edema of genital organs and colorectal cancer [25].

Evaluating the potential pathways for avian exposure to pesticides and herbicides requires careful consideration of their specific toxicity profiles to the targeted organisms [26, 27]. The unique biochemical composition of birds is believed to play a crucial role in determining the extent of exposure to pesticides and herbicides at toxicological target tissues [28]. Thus, examining the accumulation and transfer of these substances in visceral tissues of birds is essential in establishing appropriate toxicity thresholds [29, 30].

It has been recorded that avian species pose a distinctive place in the terrestrial ecosystem and are known as the best indicators of early warnings to environmental issues. Avian species, such as the Japanese quail (*Coturnix japonica*), are also considered true representative of a sound ecosystem [16, 17]. Different studies have highlighted that monitoring hematological ailments is known as a reliable and suitable biomarker because of its usefulness in identifying environmental, physiological, nutritional and pathological influences [31]. Given the growing demand for food production to support the ever-increasing human population, the use of acetochlor has become widespread. Consequently, biochemical and histopathological biomarkers in Japanese quail exposed to acetochlor have become a subject of investigation [32]. Hence, we conducted a study to investigate the impact of acetochlor exposure on the growth abnormalities, blood profile, and histopathology of various organs (testes, heart, and brain) in Japanese quail.

## Materials and methods

### Experimental design and feed management

Eighty quail were obtained from a bird market and housed in a wooden cage. All the clinically active and sexually mature male Japanese quail approximately 6 weeks of age and weighing between 115-120g were selected for this trial. All the experimental birds were housed under standard laboratory conductions including a temperature of 26–28°C, a light / dark cycle of 16/8h, and a humidity of 60–65%. After a 10-days adaptation period to laboratory conditions, the birds were randomly divided and kept into four groups (A, B, C, and D). They were provided with commercial poultry feed, which was gradually reduced over 45 days. Acetochlor (50% EC) was procured from Buraq Agro Chemicals, Punjab province Pakistan. The herbicide was mixed with corn oil and administered to all quail according to body weight using a crop tube, except for the untreated control group. Acetochlor was orally administered daily to the quail in each group, with group A serving as a control and receiving no acetochlor. Group B received 20mg/kg, group C received 30mg/kg, and group D received 40mg/kg of acetochlor. The doses of acetochlor were selected based on the basis on previously reported LD50 [11, 12] and environmental/natural concentrations reported in the literature [14, 15]. The birds were monitored daily, and tissue samples were collected on days 15, 30, and 45. No mortalities were reported. The daily feed consumption of each group was calculated, and the body weight of male Japanese quail in each group were measured weekly.

All the quail were sacrificed for collection of various tissues at different experimental intervals. All the birds were sacrificed as per the recommendations of the Animal Ethics Committee of the Islamia University of Bahawalpur and no anesthesia was used during the process of slaughtering. Briefly, we held the bird’s legs with one hand and supported the body on his upper leg. Then we grasped the head behind the skull with two fingers, quickly and firmly pulling the head down and against the knuckle of the first finger to stretch the neck and separate the skull from the atlas vertebra. Immediately after, the bird was placed in a metal killing cone (which was placed on a wall at an 180° angle, thus completely vertical) to facilitate observation of induced reflexes. After that, the skin of animals was incised along the ventral midline from the sternum to pubis and reflected back using blunt dissection to expose the peritoneal cavity.

### Hematological parameters

At 15, 30, and 45 days of the experiment, blood samples were collected from the wing vein of each bird using EDTA anticoagulant at a concentration of 2 mg/ml. Hematological parameters including erythrocyte and leukocyte counts, hematocrit, and hemoglobin concentration were measured following the previous described protocol [16, 33].

### Serum biochemistry

Throughout the study, serum was collected from the experimental bird groups at various time points, specifically on days 15, 30, and 45, by isolating the blood samples on ice. Serum biochemistry parameters, including urea, creatinine, ALT (Alanine transaminase), AST (Aspartate aminotransferase), total proteins, albumin, and plasma proteins were then measured using commercial kits and a Randox chemistry analyzer [34, 35].

### Organ weights and histopathology

Five birds were randomly selected from each group, weighed, and euthanized after blood collection. Necropsy was performed on the birds, and their testes, lungs, heart, and brain were separated, weighed, and preserved in a 15% paraformaldehyde solution. The preserved visceral organs (testes, heart, and brain) underwent histological examinations. For examination, 5μm thick slices were cut using a rotary microtome, dehydrated in alcohol, cleaned in xylene, and stained with Hematoxylin and Eosin [34, 36].

### Statistical analysis

During the course of our investigation, we presented the collected data as mean ± SE. The data obtained from each group was normally distributed and analyzed through ANOVA utilizing IBM SPSS software, version 20. Following this, a post hoc Tukey’s test was carried out at a significance level of p < 0.05 to compare the mean values (mean ± SE) of various parameters, such as serum chemistry, hematological parameters, organ weight, and histopathological parameters, between the control and experimental groups.

## Results

### Food consumption, body weight and organ weight

**Fig 1:**
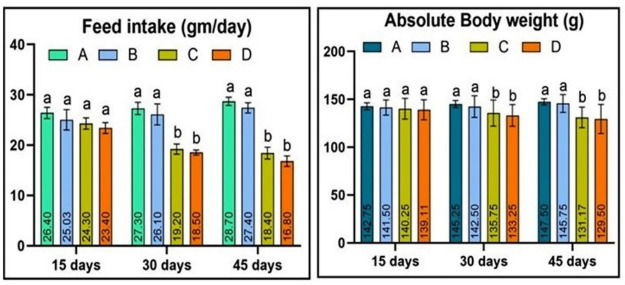
Feed intake and body weight of male Japanese quail exposed to acetochlor @ 0, 20, 30 and 40 mg/kg BW reared in groups A, B, C, and D respectively via crop tube for 45 days. Bars are indicating values (mean ±SE) while bars having different letters show significant difference (p<0.05). Data obtained from each group (mean±SE) was subjected to Turkey’s post hoc test.

**Fig 2:**
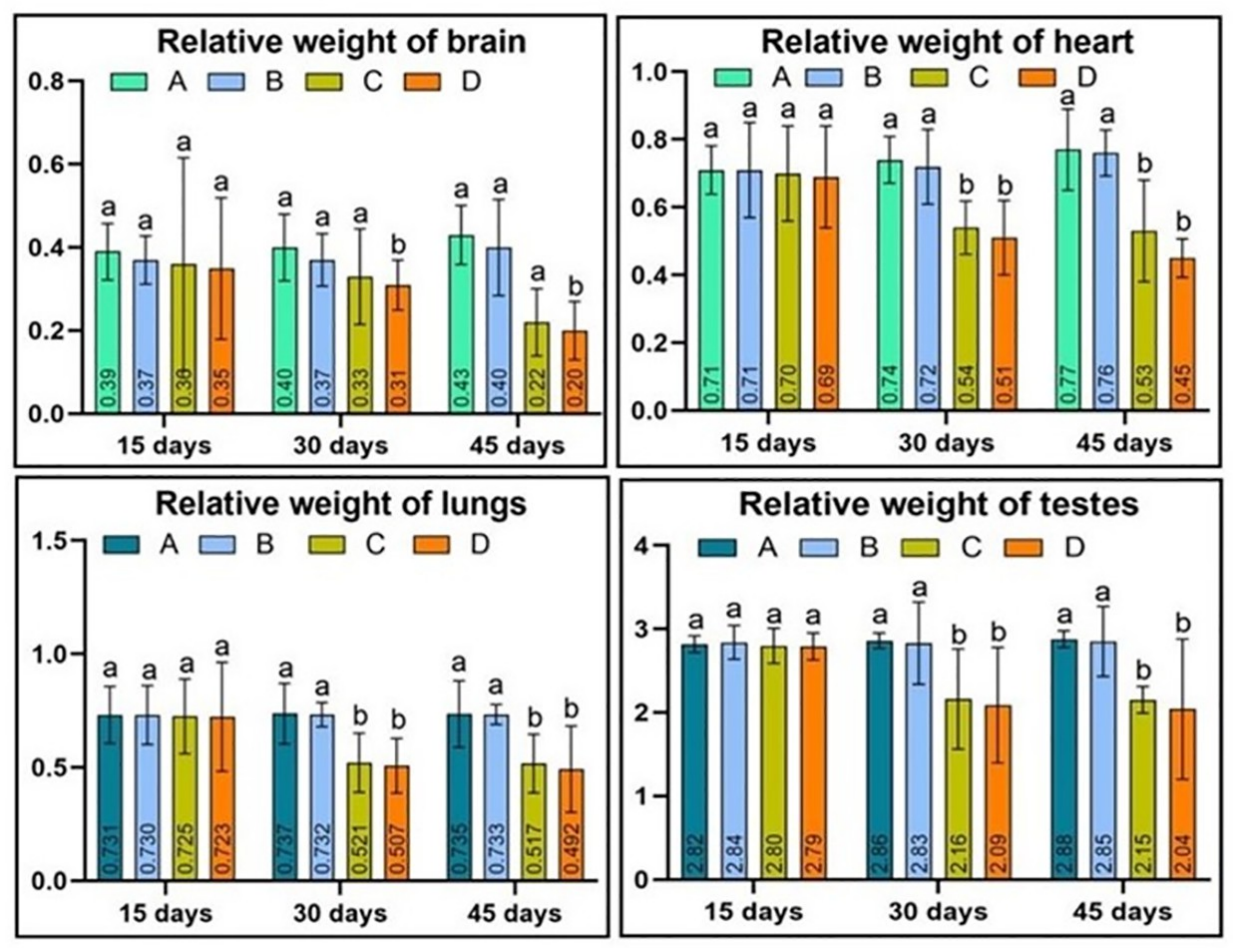
Relative weight of lungs, testes, heart, and brain of male Japanese quail exposed to acetochlor @ 0, 20, 30 and 40 mg/kg BW reared in groups A, B, C, and D respectively via crop tube for 45 days. Bars are indicating values (mean ±SE) while bars having different letters show significant difference (p<0.05). Data obtained from each group (mean±SE) was subjected to Turkey’s post hoc test.

The study found that quails administered higher doses of acetochlor (30 and 40 mg/kg) exhibited lower feed intake compared to the control group (Group A). Group B, which received a lower dose of acetochlor, did not display any significant changes in feed intake until day 45 of the experiment. However, Groups C and D showed reduced feed consumption due to the higher concentrations of acetochlor (30 and 40 mg/kg) on day 30 and 45 of the experiment, respectively. Quail exposed to higher concentrations of acetochlor also exhibited reduced body weight, while the control group and Group B, which did not receive higher doses of acetochlor, did not experience any reduction in body weight “Fig 1 and Table 1”. A notable decrease in the relative weight of the lungs, heart, and testes was observed in Group C and D on day 30 and 45 of the study, as they were treated with higher doses of acetochlor “Fig 2 and Table 2”. Furthermore, the study found that the relative weight of the brain of Japanese quail exposed to higher concentrations of acetochlor significantly decreased on day 45 of the experiment.

**Table 1:** Feed consumption of male Japanese quails exposed to various acetochlor concentrations.

Parameters and days	Groups and Treatments			
	A (control)	B (20mg/Kg)	C (30mg/kg)	D (40mg/Kg)
**Feed intake (gm/day)**				
**15**	26.4±1.11	25.03±2.01	24.3±1.13	23.4±1.07
**30**	27.3±1.23	26.1±2.09	19.2±1.03*	18.5±0.5*
**45**	28.7±0.81	27.4±1.03	18.4±1.19*	16.8±1.06*
**Body weight (g)**				
**15**	142.75±3.59	141.5±7.93	140.25±14.79	139.11±10.42
**30**	145.25±3.59	142.5±11.23	134.75±13.67*	133.25±11.29*
**45**	147.5±3.31	145.75±9.32	131.17±1.82*	129.5±5.19*

**Table 2:** Relative weight of lungs, testes, heart, and brain of male Japanese quail administered to various acetochlor concentrations.

Parameters/days	Groups/Treatments			
	A (control)	B (20mg/Kg)	C (30mg/kg)	D (40mg/Kg)
**Relative weight of lungs**				
**15**	0.731±0.126	0.730±0.13	0.725±0.164	0.723±0.24
**30**	0.737±0.134	0.732±0.053	0.521±0.13*	0.507±0.12*
**45**	0.735±0.147	0.733±0.044	0.517±0.129*	0.492±0.19*
**Relative weight of testes**				
**15**	2.82±0.10	2.84±0.70	2.80±0.71	2.79±0.163
**30**	2.86±0.092	2.83±0.49	2.16±0.60*	2.09±0.69*
**45**	2.88±0.10	2.85±0.42	2.15±0.16*	2.04±0.84*
**Relative weight of heart**				
**15**	0.71±0.071	0.71±0.14	0.70±0.24	0.69±0.15
**30**	0.74±0.069	0.72±0.170	0.54±0.079*	0.51±0.11*
**45**	0.77±0.12	0.76±0.068	0.53±0.15*	0.45±0.057*
**Relative weight of brain**				
**15**	0.39 ± 0.068	0.37±0.058	0.36±0.256	0.35±0.17
**30**	0.40± 0.08	0.37±0.063	0.33±0.115	0.31±0.06
**45**	0.43 ± 0.071	0.40±0.116	0.22±0.081*	0.20±0.07*

### Hematology

**Fig 3:**
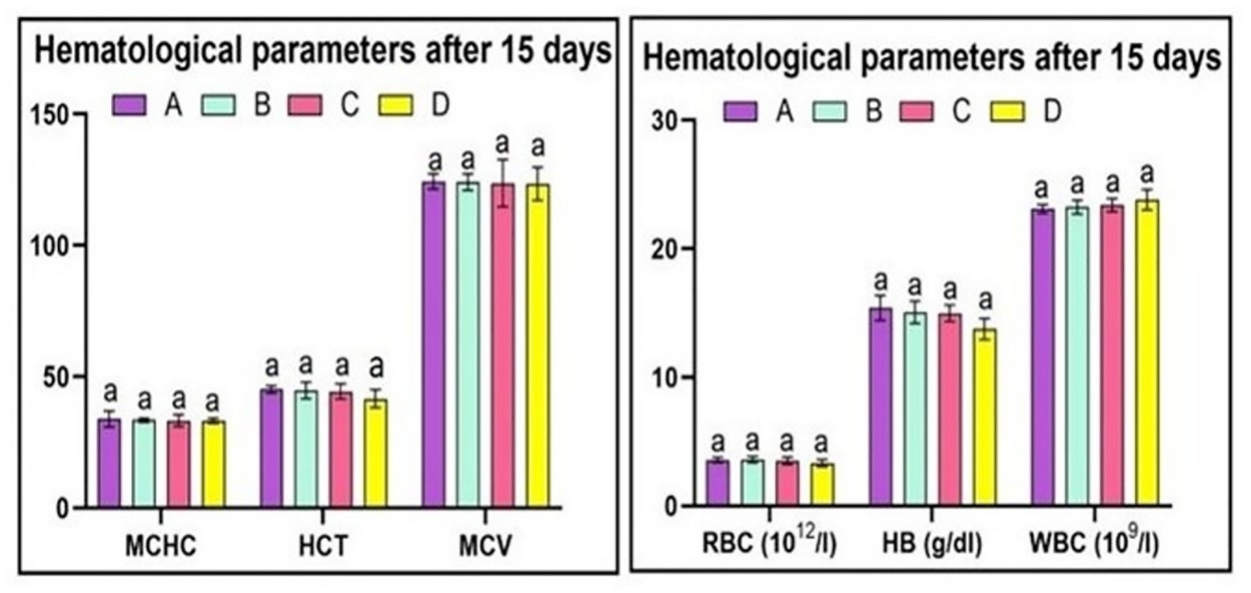
Different hematological parameters at day 15 of trial of male Japanese quail exposed to acetochlor @ 0, 20, 30 and 40 mg/kg BW reared in groups A, B, C, and D respectively via crop tube for 45 days. Bars are indicating values (mean±SE) while bars having different letters show significant difference (p<0.05). Data obtained from each group (mean±SE) was subjected to Turkey’s post hoc test.

**Fig 4:**
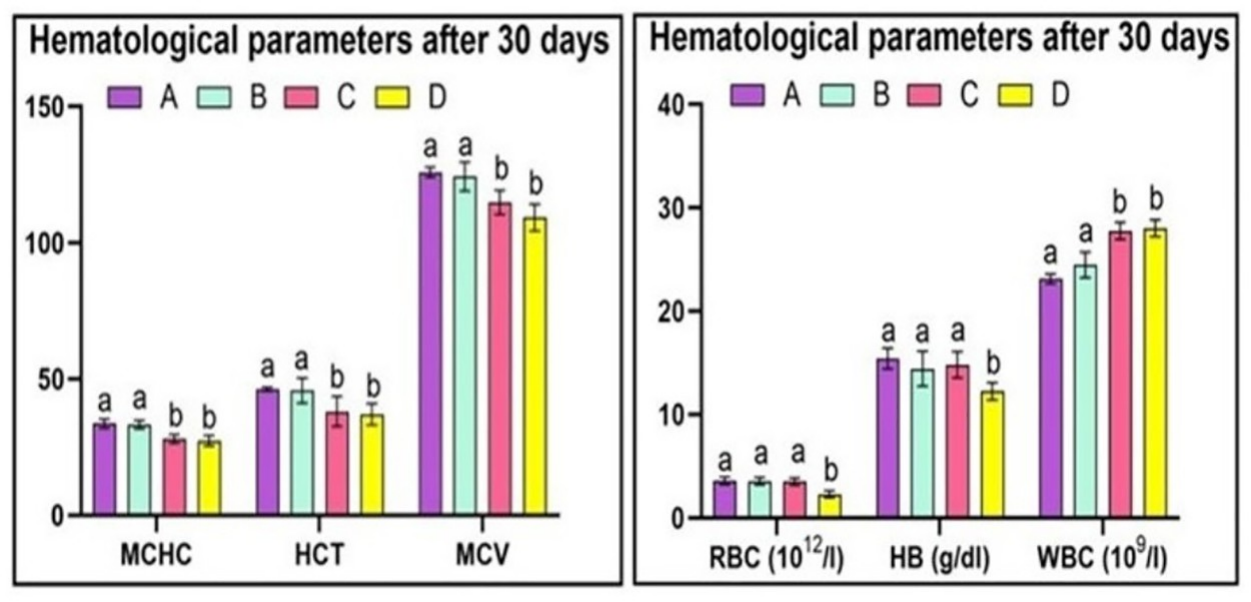
Different hematological parameters at day 30 of trial of male Japanese quail exposed to acetochlor @ 0, 20, 30 and 40 mg/kg BW reared in groups A, B, C, and D respectively via crop tube for 45 days. Bars are indicating values (mean±SE) while bars having different letters show significant difference (p<0.05). Data obtained from each group (mean±SE) was subjected to Turkey’s post hoc test.

**Fig 5:**
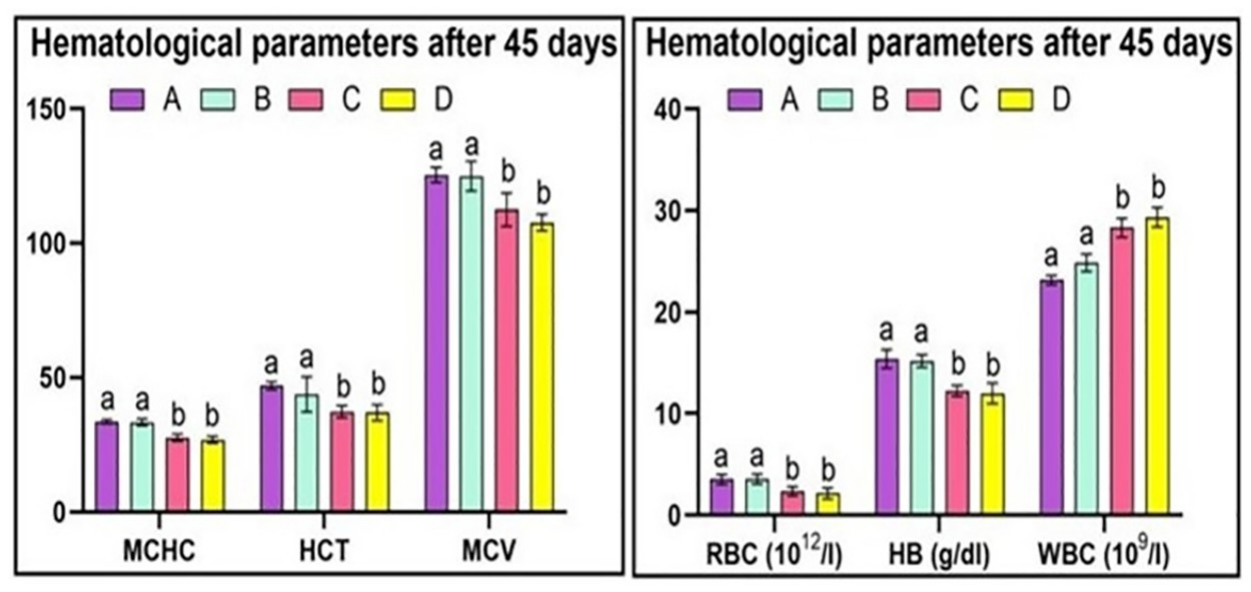
Different hematological parameters at day 45 of trial of male Japanese quail exposed to acetochlor @ 0, 20, 30 and 40 mg/kg BW reared in groups A, B, C, and D respectively via crop tube for 45 days. Bars are indicating values (mean±SE) while bars having different letters show significant difference (p<0.05). Data obtained from each group (mean±SE) was subjected to Turkey’s post hoc test.

The study revealed that the red blood cell counts in Group A quail was within normal limits. Similarly, Group B quails did not exhibit any significant changes in red blood cell count as they were exposed to a lower dose of acetochlor. However, Group C and D quails, which were given higher doses of acetochlor (30 and 40 mg/kg, respectively), showed a considerable decline in the total number of red blood cells, hemoglobin concentration, and hematocrit on day 15, 30 and 45. Hematological investigations demonstrated that red blood cell counts, hemoglobin concentration, and HCT in the control group (Group A) and the group treated with 20 mg/kg acetochlor (Group B) did not exhibit significant changes. White blood cell (WBC) count remained unchanged in the control group (Group A) and the group treated with 20 mg/kg acetochlor (Group B), while groups C and D quail demonstrated a significant rise in WBC counts on days 30 and 45 of the experiment. Our experiment revealed that there were no changes in the MCHC and MCV values in the control group (Group A) and the group treated with 20 mg/kg acetochlor (Group B). However, Groups C and D, which were treated with high doses of acetochlor (30 and 40 mg/kg, respectively), displayed a noteworthy decrease in MCV and MCHC values. The hematological changes after 15 days, 30 days and 45 days are shown in “Figs 3–5” respectively and “Table 3”.

**Table 3:** Hematology of Japanese quail treated with various acetochlor concentrations.

Parameters/days	Groups/Treatments			
	A (control)	B (20mg/Kg)	C (30mg/kg)	D (40mg/Kg)
**Red blood cell counts (10** ^**12**^ **/l)**				
**15**	3.565±0.103	3.52±0.152	3.49±0.286	3.315±0.277
**30**	3.577±0.277	3.55±0.160	3.51±0.230	2.285±0.465*
**45**	3.547±0.182	3.57±0.125	2.36±0.039*	2.155±0.541*
**HB (g/dl)**				
**15**	15.375±0.970	15.05±0.869	14.97±0.644	13.75±0.81
**30**	15.411±1.009	14.425±1.682	14.82±1.26	12.25±0.834*
**45**	15.391±0.911	15.2±2.61	12.25±0.544*	12.01±1.014*
**Hematocrit (HCT)**				
**15**	45.200±1.344	44.75±3.135	44.35±2.909	41.62±3.484
**30**	46.325±0.776	45.75±4.550	38.12±5.584*	37.050±4.034*
**45**	46.975±1.488	43.77±6.546	37.275±2.254*	36.950±3.024*
**White blood cell counts (10** ^**9**^ **/l)**				
**15**	23.11±0.34	23.25±6.53	23.41±6.514	23.82±8.038
**30**	23.14±0.49	24.47±1.23	27.79±0.835*	28.04±0.811*
**45**	23.17±0.45	24.87±0.86	28.33±0.236*	29.35±0.407*
**MCV**				
**15**	124.40±2.913	124.12±3.201	123.70±9.022	123.42±6.235
**30**	125.97±1.875	124.37±5.265	114.85±4.424*	109.32±4.882*
**45**	125.42±2.811	124.92±5.465	112.50±6.187*	107.72±3.057*
**MCHC**				
**15**	33.97±2.995	33.42±0.7588	33.32±2.247	33.27±0.903
**30**	33.65±1.687	33.35±1.621	28.02±1.530*	27.37±1.915*
**45**	33.51±0.741	33.27±1.241	27.60±1.298*	26.77±0.250*

### Serum biochemistry

**Fig 6:**
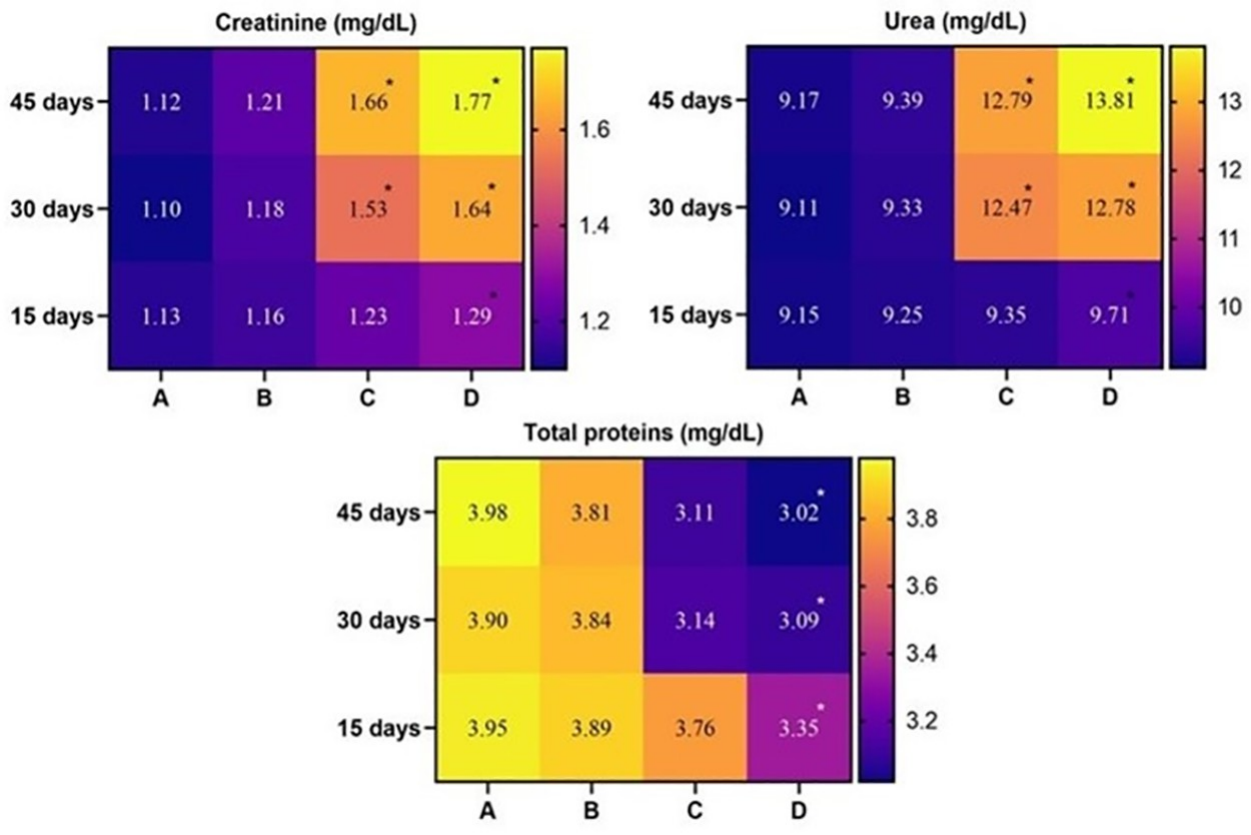
Different serum biochemical profile of male Japanese quail exposed to acetochlor @ 0, 20, 30 and 40 mg/kg BW reared in groups A, B, C, and D respectively via crop tube for 45 days. Values containing asterisks show significant difference (p<0.05). Data obtained from each group (mean±SE) was subjected to Turkey’s post hoc test.

**Fig 7:**
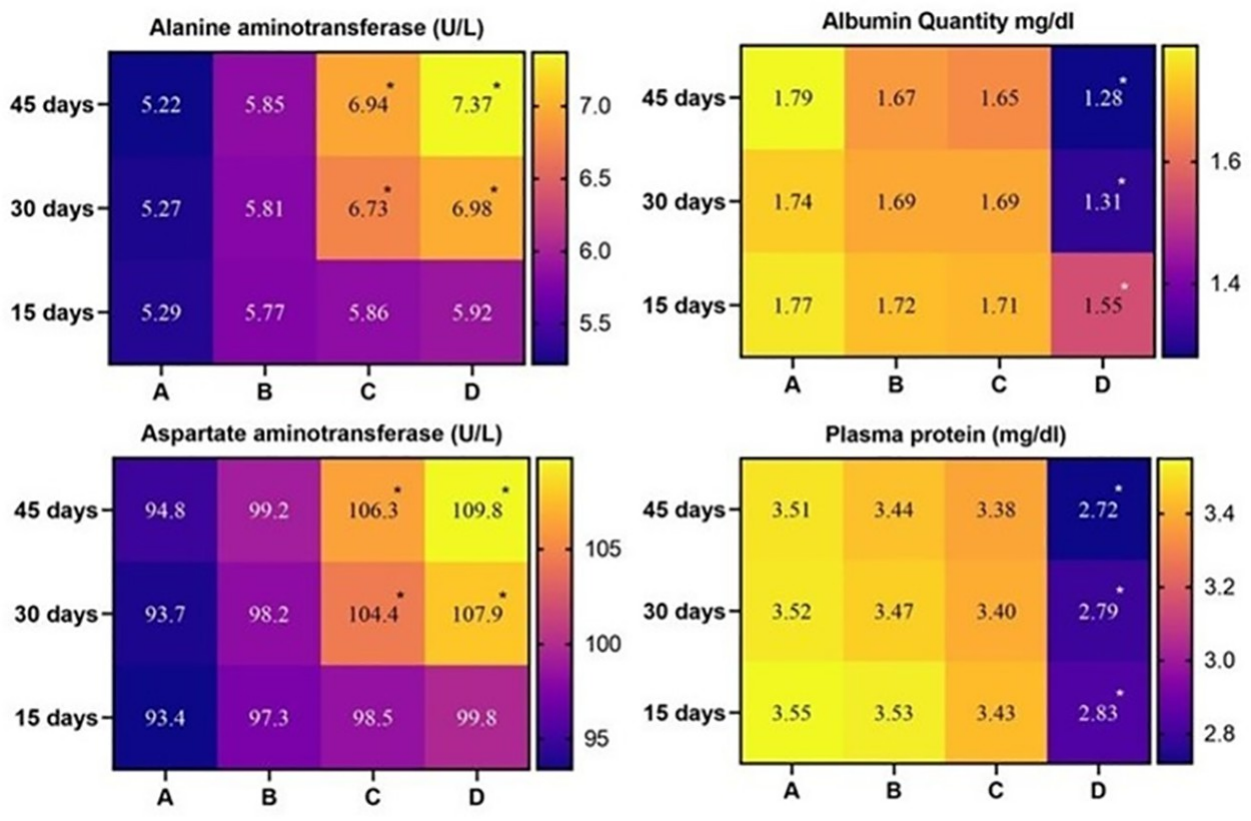
Different serum biochemical profile of male Japanese quail exposed to acetochlor @ 0, 20, 30 and 40 mg/kg BW reared in groups A, B, C, and D respectively via crop tube for 45 days. Values containing asterisks show significant difference (p<0.05). Data obtained from each group (mean±SE) was subjected to Turkey’s post hoc test.

The study showed no significant changes in kidney biomarkers (creatinine and urea) in the birds of Group A (control) and Group B (20 mg/kg acetochlor). However, the values of creatinine and urea in birds of Group C (30 mg/kg acetochlor) increased significantly on day 30 and 45 of the study. The values of creatinine and urea in birds of Group D (40 mg/kg acetochlor) significantly increased on days 15, 30, and 45 of the experiment compared to the control. Total protein, albumin, and plasma protein values in Japanese quails kept in group D (40 mg/kg acetochlor) decreased significantly on days 15, 30, and 45 of the study compared to the control “Fig 6 and Table 4”. However, there were no significant changes in total protein, albumin, and plasma protein values observed in birds of Group B and C during the study. In this experiment, the values of AST and ALT significantly increased in birds of Group C and D on days 30 and 40 of the trial compared to the control “Fig 7 and Table 4”. There were no significant changes in the values of AST and ALT observed in quails of Group B during the study.

**Table 4:** Serum biochemistry of Japanese quail exposed to various acetochlor concentrations.

Parameters/days	Groups/Treatments			
	A (control)	B (20mg/Kg)	C (30mg/kg)	D (40mg/Kg)
**Urea (mg/dL)**				
**15**	9.15±0.02	9.25±0.04	9.35±0.03	9.71±0.03
**30**	9.11±0.04	9.33±0.06	12.47±0.05*	12.78±0.04*
**45**	9.17±0.01	9.39±0.03	12.79±0.02*	13.81±0.02*
**Creatinine (mg/dL)**				
**15**	1.13±0.01	1.16±0.01	1.23±0.03	1.29±0.05
**30**	1.10±0.03	1.18±0.04	1.53±0.05*	1.64±0.03*
**45**	1.12±0.02	1.21±0.07	1.66±0.01*	1.77±0.02*
**Total proteins (mg/dL)**				
**15**	3.95±0.04	3.89±0.01	3.76±0.06	3.35±0.04
**30**	3.90±0.03	3.84±0.05	3.14±0.03*	3.09±0.01*
**45**	3.98±0.01	3.81±0.02	3.11±0.05*	3.02±0.07*
**Albumin Quantity mg/dl**				
**15**	1.77±0.03	1.72±0.02	1.71±0.01	1.55±0.02
**30**	1.74±0.05	1.69±0.04	1.69±0.06	1.31±0.04*
**45**	1.79±0.02	1.67±0.07	1.65±0.02	1.28±0.01*
**Plasma protein (mg/dl)**				
**15**	3.55±0.02	3.53±0.01	3.43±0.03	2.83±0.01
**30**	3.52±0.03	3.47±0.05	3.40±0.02	2.79±0.03*
**45**	3.51±0.06	3.44±0.04	3.38±0.05	2.72±0.04*
**Aspartate aminotransferase (U/L)**				
**15**	93.4±3.99	97.3±3.65	98.5±2.2	99.8±2.03
**30**	93.7±4.66	98.2±3.50	104.4±1.5*	107.9±1.11*
**45**	94.8±3.79	99.2±3.66	106.3±1.1*	109.8±1.32*
**Alanine aminotransferase (U/L)**				
**15**	5.29±0.07	5.77±0.04	5.86±0.10	5.92±0.12
**30**	5.27±0.09	5.81±0.06	6.73±0.13*	6.98±0.11*
**45**	5.22±0.06	5.85±0.09	6.94±0.11*	7.37±0.16*

### Gross pathology

The lungs, heart, brain, and testes of Japanese quails in Group B (20 mg/kg acetochlor) appeared almost normal in color, structure, shape, texture, and consistency upon gross examination. No tissue swelling, inflammation, or hemorrhages were observed in Group B quails compared to the control group. By day 45 of the study, the surface of the heart, lungs, brain, and testes of Group B quails remained smooth. However, the tissues of the other experimental groups (C and D) treated with higher doses of acetochlor (30 and 40 mg/kg, respectively) were affected, displayed changes in the size of the lungs, heart, brain, and testes compared to the control group.

### Testicular changes

**Fig 8:**
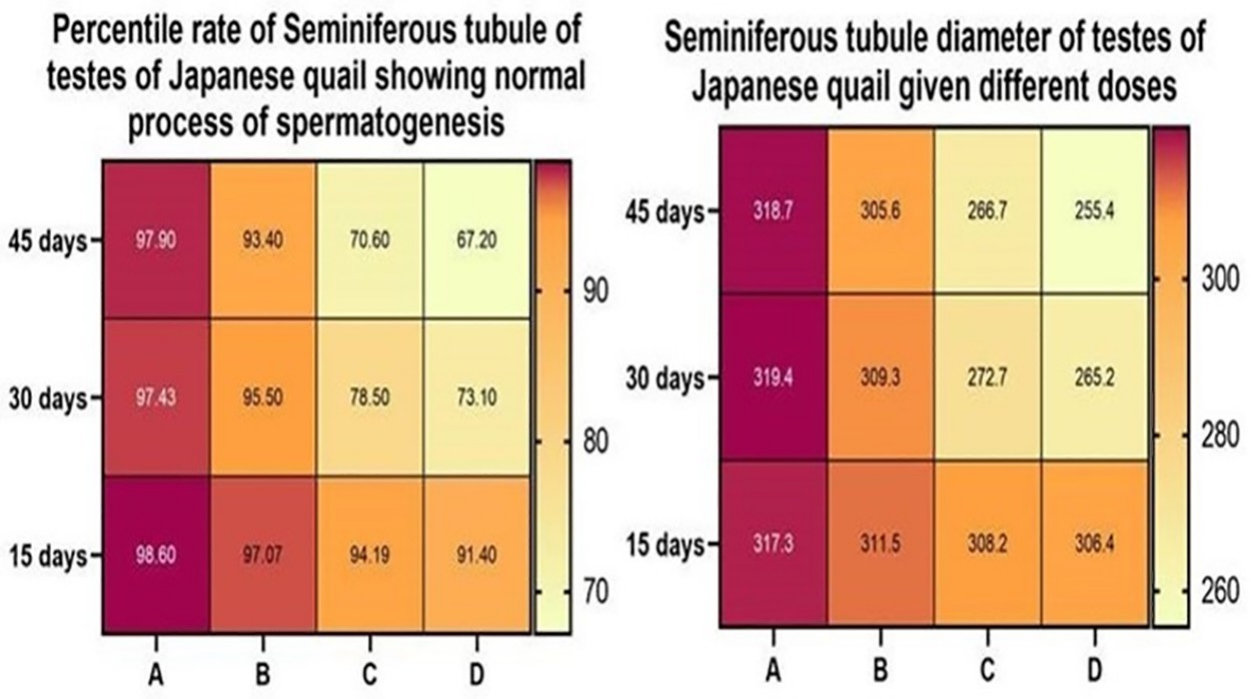
Percentage of seminiferous tubule showing normal spermatogenesis and seminiferous tubule diameter of testes of Japanese quail given different doses of acetochlor.

Treatment with acetochlor resulted in a significant decrease in the percentage rate of seminiferous tubules showing normal spermatogenesis across all treatment groups compared to the control group A. The decline was dose-dependent, with the highest dose Group D showing the largest decrease from day 15 to day 45. Similarly, there was a significant reduction in seminiferous tubule diameter in a dose-dependent manner in Groups B, C and D compared to the control group A at both day 30 and day 45. While the lowest dose group B showed a smaller decrease compared to Groups C and D as shown in “Fig 8”, the decline was still statistically significant. In contrast, control group A maintained normal levels of seminiferous tubule function and morphology throughout the 45-day experiment. Overall, environmental doses of acetochlor led to impaired testicular function and shrinkage of seminiferous tubules in a dose- and time-dependent manner in male Japanese quail. Different microscopic alterations in the testes of quail exposed to various concentrations of Acetochlor are shown in “Table 5, Fig 8”.

**Table 5:** Severity of different microscopic alterations in testes of quail exposed to different concentrations of acetochlor.

microscopic alterations	Severity level on different concentrations of Acetochlor		
	(20mg/Kg)	(30mg/kg)	(40mg/Kg)
**Pyknosis**	++	+++	++++
**Sloughed cells**	++	+++	++++
**Admixture of necrotic cells in lumen of seminiferous tubules**	++	++	+++
**Arrest of spermatogenesis**	++	++	+++
**Congestion**	++	++	+++
**Degeneration of seminiferous tubules**	+	+++	++++
**Sloughing of germinal epithelium**	++	+++	++++
**Testicular weight**	+	++	+++
**Reduction in size**	+	++	+++
**Reduction in volume**	+	++	+++

Mild (+); moderate (++); severe (+++); very severe (++++)

### Histopathology

**Fig 9:**
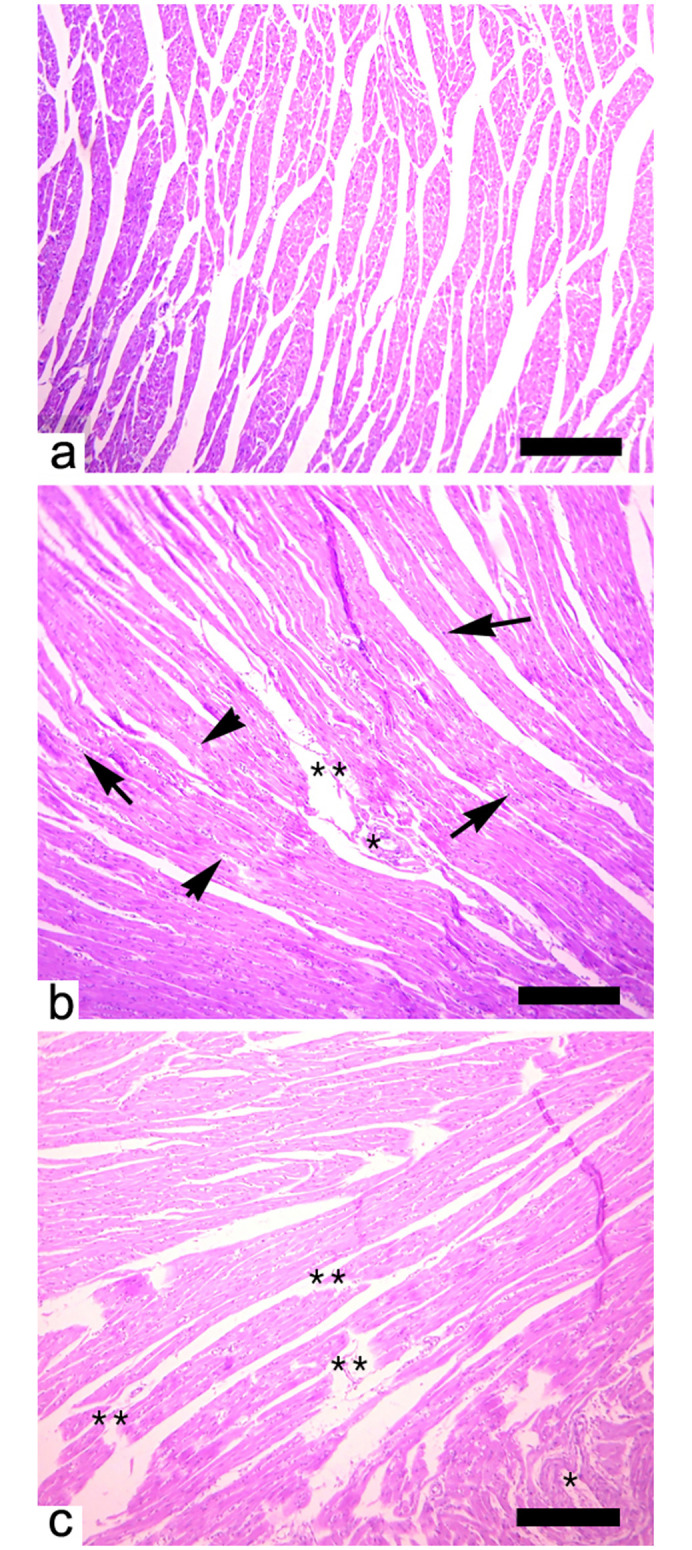
Histopathology of the heart a) showing normal histological arrangement. Heart showing various pathological lesions such as b) necrosis of cardiac cells (arrows), atrophied nuclei of cardiac myocyte (arrow heads), edema (*), detachment of epithelium of cardiac myofibers and disruption of muscle fibers (**) and c) disruption and myofibrillosis (**) and edema (*) in quail exposed to higher doses (b; 30 mg/kg) and (c;40 mg/kg) at day 45 of trial. H&E stain. X400, scale bar: 100μm.

**Fig 10:**
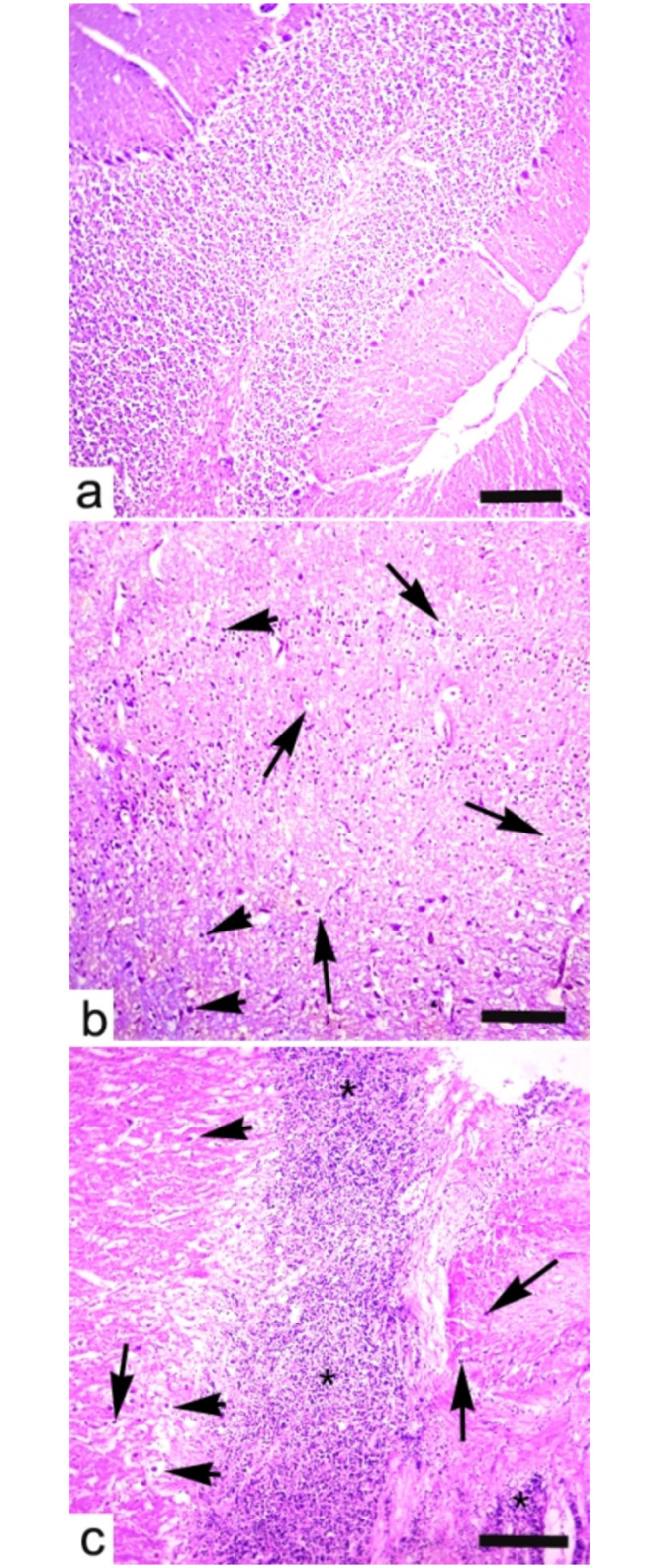
Histopathology of brain showing a) showing normal histological features of brain while b) exhibiting different pathological lesions like necrosis and degeneration of neurons (arrows), eccentric nuclei of neuron, hypertrophy of cytoplasm of neurons (arrow heads) and c) necrosis of neurons (arrows), eccentric nuclei of neurons (arrow heads) and microgliosis (*) in brain of quail exposed to higher doses (b; 30 mg/kg) and (c;40 mg/kg) at day 45 of trial. H&E stain. X400, scale bar: 100μm.

**Fig 11:**
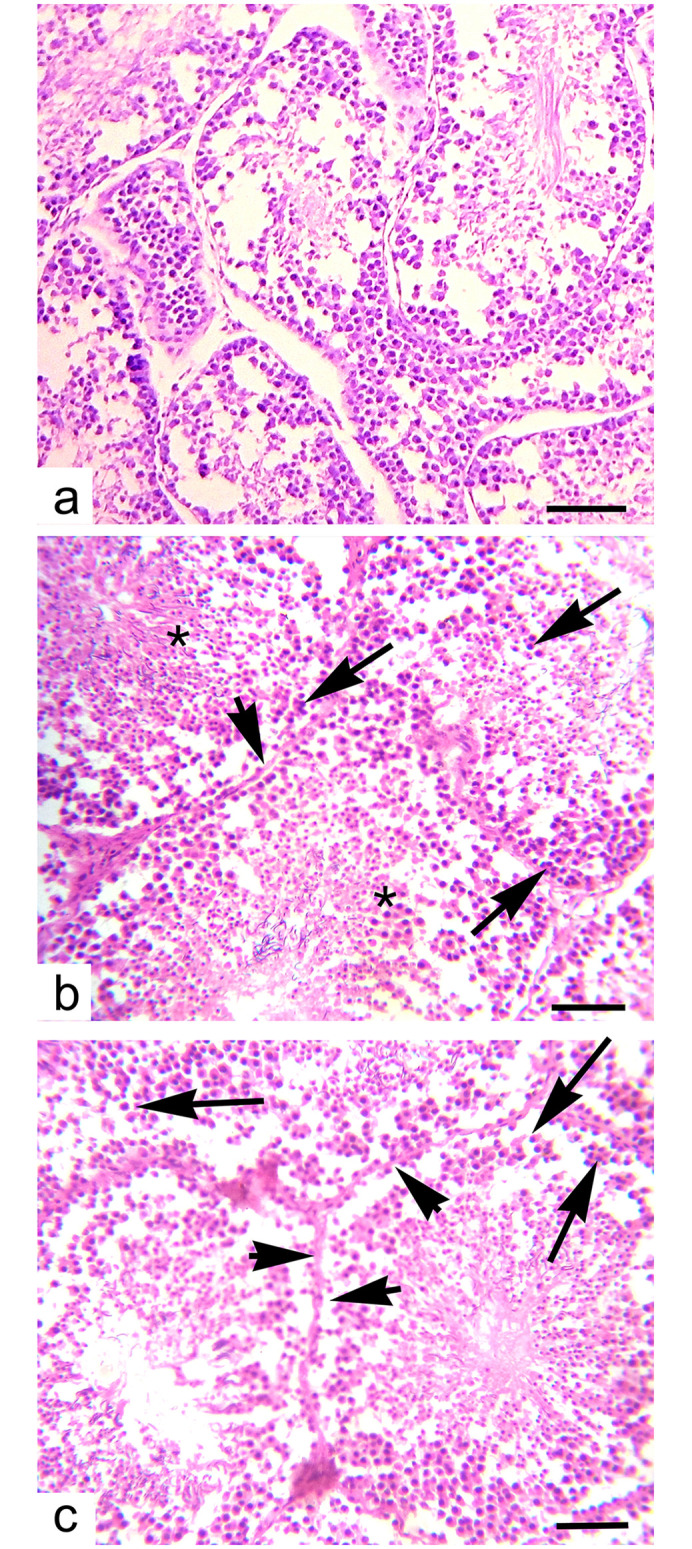
Histopathology of testes showing a) normal histological features of untreated control quail. a) different pathological lesions like admixture of necrotic cells in the lumen of seminiferous tubules (*), arrest of process of spermatogenesis (arrow head) and pyknotic nuclei (arrows) and c) arrest of process of spermatogenesis (arrow heads) and degeneration and necrosis of spermatids (arrows) in testes of quail expose to higher doses (b; 30 mg/kg) and (c;40 mg/kg) at day 45 of trial. H&E stain. X400, scale bar: 100μm.

The histopathology of heart, brain, and testes of Japanese quails in Group B (20 mg/kg acetochlor) and other experimental groups (C and D) treated with higher doses of acetochlor (30 and 40 mg/kg, respectively) displayed changes in “Figs 9–11”.

## Discussion

For the last few decades, use of natural and synthetic pesticides has significantly increased in agricultural systems to boost productivity and crop yields [22]. In recent years, concerns have escalated about the use of herbicides and their potential deleterious effects on ecosystems and human health [37]. Acetochlor is a commonly used pesticide that has been detected in soil and surface water, raising concerns about its impact on living organisms. Previous studies have demonstrated that acetochlor can cause sexual, hormonal, cardiovascular, developmental, and immunotoxic effects [38]. It is crucial to investigate the harmful effects of pesticides across various environmental compartments, including water, soil, and dust, as well as the risks associated with residues in crop production. Acetochlor is among the most widely used pesticides globally and is present in many products, including food, prompting concerns about exposure due to its widespread use for increasing production [15].

The current research did not report any deaths among the experimental groups (B-D); however, a decrease in food intake, body weight, and organ weight (testes, heart, brain, and lungs) was observed in Japanese quail administered higher doses of acetochlor (30 and 40 mg/kg). Previous studies have not provided information on the impact of acetochlor exposure on body weight, organ weight, and feed intake in quails or other avian species. Previously, decreased relative heart weight in quail exposed to pesticides has been recorded [39]. The lower relative weight of various visceral organs of quail in our study might be due to systemic effects of acetochlor. Similar to the findings of study, significantly lower feed consumption and poor body weight has also been recorded due to herbicide exposure in quail [26, 27]. The lower feed intake might be related to birds’ taste aversion, while poor body mass could be due to oxidative stress induced by acetochlor on male birds.

The present research revealed that male quails exposed to higher concentrations of acetochlor experienced a decrease in erythrocyte count, hemoglobin, hematocrit, MCV, and MCHC, whereas WBC count significantly increased at higher doses of acetochlor. In avian species, the process of hemopoiesis/erythropoiesis occurs in bone marrow (vascular sinuses). The significantly lower hematological values (erythrocytes counts, hemoglobin and hematocrit) could be related to induction of oxidative stress causing different abnormalities (myelotoxic effects and disorders in production of heme) in bone marrow of exposed quail. Furthermore, lower hemoglobin levels might be due to deleterious effects of acetochlor on maturing red blood cells and reduce values of hematocrit values might be related to decrease in the size of red blood cells in quails [16, 17, 39]. The decreased hemoglobin levels in acetochlor treated quail can also be related to oxidation of methemoglobin. The abnormal hematological profile in quail could also be linked to the disruption of hemopoietin functions in quail exposed to variable doses of acetochlor as reported in earlier studies on pesticides [26, 40]. Previous studies in birds have not explored these hematological findings specifically. However, similar results have been reported in earlier studies on fish species such as bighead carp, African catfish, and Nile tilapia, which align with the findings of the current study [5, 34, 41].

The study revealed a significant increase in kidney biomarkers (creatinine and urea), AST, and ALT in Japanese quail exposed to higher concentrations of acetochlor compared to the control group. Conversely, albumin and plasma protein values of the quails decreased significantly. Similar observations were reported in previous studies on grass carp [42] and bighead carp [34], indicating comparable findings to the present research. The increased serum biochemical profile including liver and kidneys biomarkers while lower values of serum total proteins in treated quail could be linked to induction of oxidative stress by acetochlor on hematopoietic tissues causing less supply of oxygen to multiple visceral organs resulting in free radical injuries. Previously similar facts (induction of oxidative stress) due to different herbicides including atrazine, butachlor and glyphosate have been recorded in birds [16, 17] and fish [28, 40].

There were no visible abnormalities detected in the internal organs of the birds that received treatment. However, the size and color of the lungs, heart, brain and testes of birds exposed to higher doses of acetochlor were changed to the compared to tissues of the control group.

Current research shows a significant reduction in sperm production on day 30 and 45 among birds exposed to high doses. Previously, no data were reported related to these findings in birds, but similar findings were reported in bighead carp [34].

The significantly lower blood values (hemoglobin, erythrocyte counts and hematocrit) in birds treated with higher concentrations in this study might be related to adverse effects of acetochlor on bone marrow or circulating erythrocytes. The reduction in hemoglobin levels in birds could be due to reduced feed consumption, a lower amount of hemopoietin and poor performance of hemopoietic organs, while decreased hematocrit values can be related to destruction of red blood cells [17, 43, 44]. The histopathological ailments in the testicular tissues of quail exposed to higher quantities of herbicides, such as arrested spermatogenesis, necrotic cells, detachment of seminiferous tubular epithelium, and the presence of a mixture of necrotic cells in lumen of seminiferous tubules, might be due to impaired mitosis [16, 17]. The testicular ailments in this study can be related to over release of IL-1a and IL-33 from damaged cells resulting in transcription of various cytokine genes and release of extracellular (biglycan and hyaluronan) and intracellular (HsP, neuropeptides, and N formal peptides) DAMPS. No report could be found about the toxicity of acetochlor on various visceral organs of quail in published literature. However, various intestinal histopathological ailments (inflammation, cellular anomalies, loss of tight junctions and barrier disruption) due to acetochlor in broiler birds have been observed [45]. It is recorded that acetochlor induced pathological lesions in intestine via stimulation of different pathways (TNFα/TNFR1 and TLR4/NF-κB/NLRP3) initiating RIPK1/RIPK3 complex formation, activation of Caspase-1 and NLRP3 inflammasomes, and MLKL phosphorylation [45]. In the current study, different necrotic ailments in various visceral organs including testes, heart and brain might be related to over release of IL-33 and IL-1α from necrotic cells [45, 46]. Moreover, the necrotic alterations in multiple tissues of quail in this study including, heart, testes and brain could also be related to the induction of oxidative stress by acetochlor causing over-generation of free radicals leading to injuries to cellular/biological membranes. Previously, histologically, degeneration of muscle fibers of heart and loss of striations have been in heart of quail exposed to pesticides [39]. Histologically, the testicular changes like necrosis of spermatids and decreased process of spermatogenesis in acetochlor-treated quail at higher doses might be related to toxic impacts on maturating germinal epithelium during mitosis as reported in an earlier study due to pesticide in birds [47]. It is tempting to speculate that the histopathological ailments in multiple tissues of acetochlor-treated quail in present study may be due to acetylation of histone, methylation of DNA material, nitration of genomic proteins, oxidation of mRNA and over-release of apoptotic factors from mitochondria as a result of induction of oxidative stress [27, 47].

## Conclusion

This study elucidates the dose-dependent toxicological effects of the herbicide acetochlor in male Japanese quails (*Coturnix japonica*). Acetochlor exposure induced measurable declines in feed consumption, body mass, and relative organ weights, along with deteriorations in hematologic indices in a concentration-dependent manner. Additionally, dose escalations precipitated elevations in clinical chemistry markers indicative of renal and hepatic injury alongside macroscopic and presumed histopathological damage within cardiac, pulmonary, neural, and reproductive tissues. The magnified severity of clinical manifestations, hematotoxicity, clinical pathology alterations, and gross organ pathologies with increasing acetochlor concentrations is highly suggestive of systemic, oxidative, and histotoxic modes of action. Cumulatively, the data demonstrates acetochlor’s potential to inflict dose-responsive, multi-organ toxicity in male Japanese quails. Further toxicological assessments are warranted to establish no-observed-adverse-effect-levels and determine safety margins to better guide evidence-based regulation of agricultural acetochlor usage to mitigate ecological risk, particularly in avian species that may exhibit greater susceptibility.
